# Neonatal diet and growth outcomes in hospitalised very preterm infants: an observational study in middle income countries in Africa, Asia, and Latin America

**DOI:** 10.7189/jogh.15.04340

**Published:** 2025-12-22

**Authors:** Mandy B Belfort, Danielle EY Ehret, Lucy T Greenberg, Anne CC Lee, Renato S Procianoy, Katherine EA Semrau, Rita C Silveira, Lloyd Tooke, Erika M Edwards

**Affiliations:** 1 Harvard Medical School and Brigham and Women’s Hospital, Department of Pediatrics, Boston, Massachusetts, USA; 2 Vermont Oxford Network, Burlington, Vermont, USA; 3 University of Vermont Robert Larner, MD College of Medicine, Department of Pediatrics, Burlington, Vermont, USA; 4 University of Vermont College of Engineering and Mathematical Sciences, Department of Mathematics and Statistics, Burlington, Vermont, USA; 5 Warren Alpert Medical School of Brown University, Brown Global Alliance of Infant and Maternal Health Research, Department of Pediatrics, Providence, Rhode Island, USA; 6 Universidade Federal do Rio Grande do Sul and Hospital de Clinicas de Porto Alegre, Department of Pediatrics, Neonatal Section, Porto Alegre, Rio Grande do Sul, Brazil; 7 Harvard T.H. Chan School of Public Health and Brigham and Women’s Hospital, Ariadne Labs, Boston, Massachusetts, USA; 8 University of Cape Town, Department of Paediatrics and Child Health, Cape Town, South Africa

## Abstract

**Background:**

Up-to-date data from middle-income countries are needed to inform in-hospital feeding practices among small, vulnerable newborns. We aimed to quantify growth indicators and their associations with in-hospital diet for infants born very preterm or with a very low birth weight (VLBW) of 401–1500 g in 12 middle income countries in Africa, Asia, and Latin America.

**Methods:**

We performed an observational cohort study of infants born at 22–29 weeks’ gestation or VLBW from 2018 to 2024 among Vermont Oxford Network member hospitals in 12 middle income countries. We used linear regression to estimate adjusted mean change in weight and head circumference (z-scores based on Fenton reference) from birth to hospital discharge by category of enteral diet at discharge/transfer (human milk only, mixed (human milk with formula and/or fortifier), and formula only), adjusting for confounders, in the entire cohort and stratified by birth weight and foetal growth status.

**Results:**

Among 35 843 infants, the median length of stay was 50 days (interquartile range = 37, 65). Eighty-four percent were receiving at least some human milk at discharge (34% human milk only, 50% mixed, 16% formula only). Adjusted mean weight z-score declined by 1.40 in the human milk only group, 1.32 in the mixed group, and 1.17 in the formula only group. The adjusted estimated difference between mixed diet and formula only groups was 0.15 z-scores (95% confidence interval (CI) = 0.10, 0.20), and between the mixed and human milk only groups was 0.08 (95% CI = 0.00, 0.15). Head growth differed little between groups.

**Conclusions:**

Human milk use was high at discharge in this vulnerable population of infants born at 22–29 weeks or VLBW in middle- income countries. Infants fed a mixed diet gain weight more slowly than infants fed only formula. Rigorous intervention studies are needed determine optimal nutrient delivery strategies for infants fed human milk in this context.

Human milk is recommended for virtually all newborn infants worldwide due to short- and long-term health benefits for both infants and mothers [1]. Global efforts have increasingly focussed on improving the survival of small, vulnerable newborns, meaning those born preterm (<37 weeks’ gestation); small for gestational age (SGA), *i.e.* with a birth weight below the tenth percentile; and/or with low birth weight (<2500 g) [2]. Within this category of small, vulnerable newborns, those born at very preterm gestations and/or at a very low birth weight (VLBW) of <1500 g are at heightened risk for mortality and for serious medical complications related to their immaturity and small size, for which human milk has protective effects.

Feeding represents a major aspect of in-hospital medical care for small, vulnerable newborns. Given strong and consistent evidence about their health benefits, global World Health Organization (WHO) recommendations emphasise the use of maternal milk and the avoidance of formula for all infants [1]. Specific to small, vulnerable newborns, maternal milk protects against necrotising enterocolitis (NEC) [3], a life-threatening gastrointestinal disorder that affects preterm infants almost exclusively. A major clinical challenge lies in maximising the use of human milk while also meeting daily nutrient intake requirements, which are substantially higher for very preterm and VLBW infants than for full-term infants [4]. These high nutrient requirements are difficult or impossible to meet with human milk alone when fed at standard volumes for preterm infants [4]. Commercial human milk fortifiers provide protein, energy, and micronutrients with minimal displacement of milk volume [5], but product availability is often limited in low- and middle-income countries [6], where infant formula may be used as an alternative [7]. However, up-to-date data about neonatal intensive care unit (NICU) feeding practices in middle-income countries are limited [8–12].

Physical growth of very preterm infants during the NICU hospitalisation indexes nutrient accretion into tissues during the critical period in development that normally occurs during the third trimester of gestation. Data from the USA and the UK indicate that, while NICU weight gain and head growth of very preterm and VLBW infants lag behind the foetus in utero, in-hospital growth has improved over the past two decades [13,14]. A few studies have examined growth outcomes among very preterm, VLBW infants in low- or middle-income countries, with most suggesting slower in-hospital growth as compared with high income countries [8–12,15]. Yet comprehensive data and analyses of temporal trends from these contexts are limited.

We aimed to address knowledge gaps regarding NICU growth outcomes and feeding practices for very preterm infants in middle income countries by leveraging an existing large quality improvement database. Our goal was to examine in-hospital use of human milk only, mixed, and formula only diets, and outcomes of weight gain and head growth from birth to discharge or transfer, overall and by diet type.

## METHODS

### Population

We used all available data from international sites participating in the Vermont Oxford Network (VON), a USA-based voluntary, non-profit collaborative professional organisation focussed on neonatal health care quality improvement and safety worldwide. Member hospitals participating from both the public and the private sector participating in the VLBW database collect and submit data from all infants of 22–29 weeks’ gestation or 401–1500 g birth weight who are inborn or admitted to the hospital within 28 days of birth. Infants from member hospitals meeting either the gestational age or the birth weight criteria, or both, are included in the VLBW database. Member hospitals also completed annual surveys about services offered and other hospital-level information. All data collection followed a detailed manual of operations for standardised data collection and entry [16]. Outcomes of VLBW infants from middle income countries participating in VON have been published elsewhere [17].

For this analysis, we included infants from the VON VLBW database who were born at 22–29 completed weeks’ gestation between 2018 and 2024 in 12 upper-middle and lower-middle income countries, as defined by the World Bank [18]. The number of member hospitals and number of participating infants varied by country (Table S1 in the **Online Supplementary Document**): Argentina, 6 hospitals; Botswana, 2; Brazil, 57; China, 2; Columbia, 2; India, 30; Malaysia, 2; Mexico, 1; Namibia, 2; Pakistan, 1; South Africa, 115; Turkey, 2. The initial sample across these 222 hospitals comprised 44 382 infants, of whom we excluded 1221 with congenital anomalies; 3498 infants who were >42 weeks’ postmenstrual age (PMA) at discharge; 1907 who had an initial length of stay of <15 days; 885 who did not receive enteral feeding at discharge; 395 who were transferred to another hospital for a reason other than growth, such as medical or diagnostic services or surgery; and 633 with missing data. This left a sample of 35 843 infants for analysis (Figure S1 in the **Online Supplementary Document**).

### Measures

Local hospital staff collected all data from medical records following the VON manual of operations [16]. Body weight and head circumference from birth and discharge/transfer were converted to z-scores for PMA and sex using the Fenton reference, which was created through a meta-analysis of population-based birth cohorts from six high income countries (Germany, USA, Italy, Australia, Scotland, and Canada). Those birth data were harmonised with WHO Growth Standard to provide smoothed reference curves covering from 22–50 weeks of PMA [19]. For our primary indicators of growth, we calculated the change in z-score from birth to discharge/transfer to indicate growth relative to the foetal reference as recommended [20]. We also calculated weight gain velocity using an exponential model [21] and the weekly average rate of absolute head growth as ((head circumference at discharge/transfer minus head circumference at birth)/length of stay). The VON does not collect data on body length.

Using VON established data definitions [20], we categorised enteral diet within 24 hours of discharge or transfer as: human milk only, meaning unfortified milk as the only enteral feeding; mixed, meaning human milk in combination with either fortifier or formula; or formula only. Available data did not allow us to discern maternal milk from pasteurised donor human milk, nor were we able to differentiate human milk fortifier from formula in the mixed diet group. Diet data from earlier in the hospitalisation were not collected.

Covariates included potential confounders such as gestational age, birth weight z-score, SGA (birth weight <10th percentile for gestational age), sex, multiple gestation, PMA at discharge/transfer, birth hospital, and neonatal diagnoses of NEC defined by clinical and radiographic features [16] and chronic lung disease defined as receiving supplemental oxygen at 36 weeks’ PMA or discharged/transferred on supplemental oxygen from 34–35 weeks’ PMA. We also grouped countries by region (Africa, Asia, and Latin America).

### Statistical analysis

We examined the distributions of diet categories and growth indicators (weight and head z-score change, weight gain velocity, head growth), overall and by region. For continuous variables, we report medians and interquartile ranges (IQRs) because some variables, such as birth weight z-score, were slightly skewed. We used a linear regression model to estimate marginal means of each outcome by diet category, adjusting for potential confounders (sex, gestational age at birth, birth weight z-score, PMA at discharge, NICU morbidity) and accounting for clustering within hospitals with generalised estimating equations. We chose the generalised estimating equations method with robust variance estimator to account for individuals clustered within hospitals and used an exchangeable correlation structure because we expect individuals within the same hospital to share common unmeasured factors, leading to similar correlation between all individuals in the same hospital. We ensured that linear regression assumptions were met, including normally distributed residuals. We compared these adjusted means among the three diet categories (human milk only, mixed, formula only), using the mixed diet group as the reference. We used the Bonferroni correction to define statistical significance due to multiple comparisons. To evaluate potential effect modification, we stratified by gestational age category (≤26 weeks, 27–29 weeks, 30–32 weeks) and foetal growth category (SGA *vs*. not) and fit models with interaction terms. We hypothesised that infants who were more immature at birth would experience overall slower growth compared to more mature infants, due to their greater illness severity, higher nutrient requirements, and more frequent feeding intolerance. We further hypothesised that more immature infants would experience more pronounced differences in discharge size across diet categories. To examine trends in growth from 2018 to 2024, we plotted adjusted mean growth variables by year and tested significance by adding a linear time trend to adjusted linear regression models, overall and stratified by region (Africa, Asia, Latin America). We used *R*, version 4.2.2 (R Core Team, Vienna, Austria) and SAS, version 9.4 (SAS Institute Inc., Cary, North Carolina, USA) for data analysis.

## RESULTS

Of the 35 843 participants, 44% were from Africa, 9% from Asia, and 47% from Latin America (**Table 1**; Table S1 in the **Online Supplementary Document**). Median gestational age was 29.6 years (IQR = 28.0, 31.0) and birth weight was 1180 grams (IQR = 995, 1348). Median birth weight z-score was −0.39 (IQR = −0.95, 0.18) and 13% were SGA. Median length of stay was 50 days (IQR = 37, 65). At discharge/transfer, median PMA was 36 completed weeks (IQR = 35, 38) and 34% were receiving only human milk, 50% were receiving a mix of human milk and formula and/or fortifier, and 16% were receiving formula only. Median weight z-score for PMA at discharge/transfer was −1.73 (IQR = −2.41, −1.11) and head circumference z-score was −0.92 (IQR = −1.66, −0.23). Five percent experienced NEC and 13% experienced chronic lung disease. At a hospital level, of 222 hospitals, 174 (78%) were private hospitals, the median number of NICU beds was 10 (IQR = 7, 15), and 219 (99%) reported using parenteral nutrition.

**Table 1:** Characteristics 35 843 very preterm infants born between 2018 and 2024 in 12 middle-income countries*

		Diet at discharge or transfer			
	**Overall (n = 35 843)**	**Human milk only (n = 12 255, 34%)**	**Mixed (n = 17 966, 50%)†**	**Formula only (n = 5622, 16%)**
**Infant characteristics at birth**				
Region				
*Africa*	15 754 (44)	7893 (64)	5163 (29)	2698 (48)
*Asia*	3155 (9)	949 (8)	1995 (11)	211 (4)
*Latin America*	16934 (47)	3413 (28)	10808 (60)	2713 (48)
Gestational age, weeks	29.6 (28.0, 31.0)	29.9 (28.0, 31.0)	29.7 (28.0, 31.0)	29.0 (28.0, 30.6)
Birth weight, grams	1180 (995, 1348)	1200 (1020, 1360)	1175 (985, 1340)	1140 (956, 1320)
Birth weight z-score‡	−0.39 (−0.95, 0.18)	−0.35 (−0.92, 0.21)	−0.44 (−1.00, 0.14)	−0.32 (−0.87, 0.26)
Small for gestational age§	4566 (13)	1371 (11)	2574 (14)	621 (11)
Birth head circumference in cm, MD (IQR)‡	27.0 (25.5, 28.0)	27.0 (26.0, 28.5)	27.0 (25.5, 28.0)	27.0 (25.0, 28.0)
Birth head circumference z-score, MD (IQR)	0.10 (−0.64, 0.84)	0.24 (−0.53, 0.99)	0.02 (−0.71, 0.73)	0.19 (−0.60, 0.98)
Gestational age category in completed weeks				
*≤26*	3288 (9)	942 (8)	1622 (9)	724 (13)
*27–29*	16026 (45)	5345 (44)	8005 (45)	2676 (48)
*30–32*	16529 (46)	5968 (49)	8339 (46)	2222 (40)
Male sex	17192 (48)	5848 (48)	8601 (48)	2743 (49)
Multiple gestation	8448 (24)	2237 (18)	4829 (27)	1382 (25)
**Infant characteristics at discharge or transfer**				
Postmenstrual age in weeks, MD (IQR)	36.7 (35.3, 38.3)	36.3 (35.0, 37.6)	36.9 (35.6, 38.3)	37.4 (36.0, 39.3)
Chronological age in days, MD (IQR)	50 (37, 65)	46 (34, 60)	50 (37, 66)	58 (43, 74)
Weight in grams, MD (IQR)	2020 (1800, 2305)	1880 (1750, 2085)	2082 (1850, 2390)	2145 (1925, 2480)
Weight z-score, MD (IQR)‡	−1.73 (−2.41, −1.11)	−1.85 (−2.53, −1.26)	−1.66 (−2.32, −1.03)	−1.72 (−2.45, −1.02)
Head circumference in cm	31.5 (30.0, 33.0)	31.0 (30.0, 32.0)	31.5 (30.0, 33.0)	32.0 (31.0, 33.0)
Head circumference z-score, MD (IQR)‡	−0.92 (−1.66, −0.23)	−0.92 (−1.65, −0.19)	−0.92 (−1.65, −0.24)	−0.91 (−1.72, −0.16)
Necrotizing enterocolitis¶	1687 (5)	521 (4)	834 (5)	332 (6)
Chronic lung disease¶	4705 (13)	966 (8)	2681 (15)	1058 (19)
NEC and/or CLD	5972 (17)	1389 (11)	3288 (18)	1295 (23)

CLD – chronic lung disease, IQR – interquartile range, MD – median, NEC – necrotising enterocolitis

*Presented as n (%) unless specified otherwise.

†Indicates human milk plus formula and/or fortifier.

‡Fenton 2013 reference.

§Defined as birth weight below the tenth percentile on the Fenton reference [16].

¶Defined in Vermont Oxford Network 2021 Manual of Operations [16].

After adjustment, we observed a less pronounced decline in weight z-score among infants fed formula only as compared with a mixed diet (adjusted mean z-score change, −1.32 for mixed and −1.17 for formula only; estimated difference 0.15 z-scores, 95% confidence interval (CI) = 0.10, 0.20). We observed a similar pattern for weight gain velocity, with faster weight gain velocity in the formula only group (adjusted mean, 11.9 g per kg/day) compared with the mixed diet group (adjusted mean, 11.3 gram per kg/day), estimated difference 0.15 g per kg/day (95% CI = 0.10, 0.20) (**Table 2**, **Table 3**).

**Table 2:** Continuous growth indicators at discharge, overall and stratified by GA and foetal growth status and region

		x̄ (SE)			
	**n**	**Weight z-score change**	**Weight gain velocity in g per kg/day**	**Head z-score change**	**Head growth in cm/week**
**Overall**	35843	−1.43 (0.04)	10.84 (0.34)	−1.06 (0.05)	0.61 (0.01)
**GA, completed weeks**					
≤26	3288	−1.91 (0.06)	11.87 (0.17)	−1.59 (0.07)	0.66 (0.01)
27-29	16026	−1.56 (0.05)	10.9 (0.33)	−1.25 (0.06)	0.61 (0.01)
30-32	16529	−1.2 (0.04)	10.57 (0.38)	−0.78 (0.04)	0.59 (0.01)
**Foetal growth status**					
SGA*	4566	−1.25 (0.05)	12.8 (0.31)	−0.5 (0.06)	0.66 (0.01)
Not SGA	31277	−1.45 (0.04)	10.55 (0.34)	−1.14 (0.05)	0.6 (0.01)
**Region**					
Africa	15754	−1.58 (0.05)	9.85 (0.51)	−1.21 (0.07)	0.57 (0.02)
Asia	3155	−1.29 (0.07)	10.78 (0.48)	−1.21 (0.11)	0.55 (0.03)
Latin America	16934	−1.32 (0.06)	11.77 (0.21)	−0.91 (0.07)	0.65 (0.01)

GA – gestational age, SGA – small for gestational age, SE – standard error, x̄ – mean

*Defined as birth weight below the tenth percentile on the Fenton reference.

**Table 3:** Unadjusted and adjusted associations of diet at discharge or transfer with weight gain and head growth (n = 35 843)

	x̄ (SE)			Difference (95% CI)	
**Growth measure**	**Human milk only diet, 12 255 (34%)**	Mixed diet, 17 966 (50%)*	**Formula only diet, 5622 (16%)**	**Human milk only diet *vs*. mixed diet**	**Mixed diet *vs*. formula only diet**
**Unadjusted**					
Weight z-score change	−1.59 (0.04)	−1.30 (0.05)	−1.48 (0.06)	0.29 (0.19, 0.38)†	−0.18 (−0.28, −0.08)†
Weight gain velocity in g per kg/day	9.92 (0.29)	11.32 (0.45)	11.29 (0.31)	1.41 (0.70, 2.11)†	−0.03 (−0.84, 0.78)
Head z-score change	−1.17 (0.06)	−0.98 (0.07)	−1.09 (0.05)	0.19 (0.05, 0.32)†	−0.10 (−0.22, 0.01)
Head growth in cm/week	0.57 (0.01)	0.62 (0.02)	0.63 (0.01)	0.05 (0.01, 0.08)†	0.01 (−0.02, 0.04)
**Adjusted**‡					
Weight z-score change	−1.40 (0.03)	−1.32 (0.04)	−1.17 (0.03)	0.08 (0.00, 0.15)	0.15 (0.10, 0.20)†
Weight gain velocity in g per kg/day	11.08 (0.16)	11.30 (0.18)	11.93 (0.13)	0.22 (−0.23, 0.66)	0.63 (0.35, 0.91)†
Head z-score change	−1.15 (0.04)	−1.13 (0.04)	−1.05 (0.04)	0.02 (−0.05, 0.08)	0.08 (0.01, 0.16)
Head growth in cm/week	0.58 (0.01)	0.59 (0.01)	0.61 (0.01)	0.01 (−0.01, 0.02)	0.02 (0.00, 0.03)

CI – confidence interval, SE – standard error, x̄ – mean

*Human milk plus formula or fortifier.

†Estimates adjusted for sex, gestational age at birth, birth weight z-score, postmenstrual age at discharge, NICU morbidity, hospital.

‡Difference is statistically significant after Bonferroni correction (*P* < 0.025).

We observed differences in weight outcomes between formula only and mixed diets across all strata of gestational age (<27, 27–29, and 30–32 weeks) and SGA status (Table S2 in the **Online Supplementary Document**). We found statistically significant interactions of diet and gestational age category with weight outcomes (*P*-value for interaction <0.001), but not head growth, and of diet and foetal growth category with all growth indicators (*P*-value for interaction <0.001).

**Figure 1:**
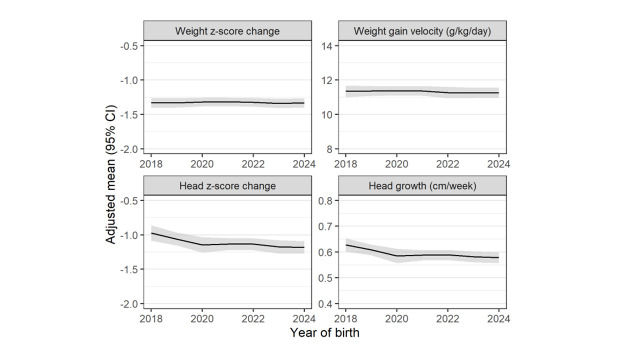
Growth outcomes over time in the overall cohort. Weight z-score change (*P* = 0.93) and weight gain velocity (*P* = 0.53) did not change over time, while head z-score change (*P* = 0.001) and head growth in cm/week (*P* < 0.001) both declined over time.

**Figure 2:**
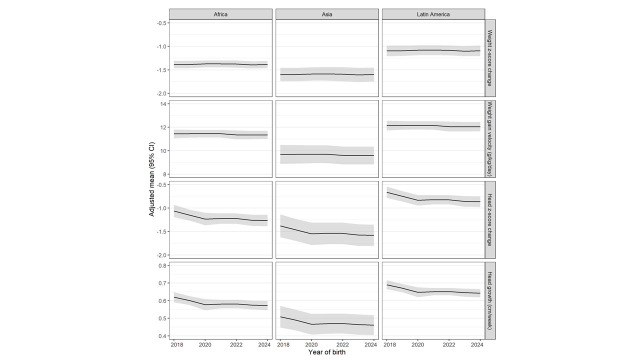
Growth outcomes over time by region. Linear trends were statistically significant, indicating declining head z-score change (*P* = 0.004) and declining head growth (*P* = 0.003) in Latin America.

Linear trends for changes in growth outcomes over time (**Figure 1**) and by region (**Figure 2**) were statistically significant, indicating declining head z-score change overall (linear time trend *P* < 0.001) and declining head growth in cm/week overall (*P* < 0.001) and in Latin America (*P* = 0.04 and *P* = 0.003). Diet at discharge varied by region (Figure S2 in the **Online Supplementary Document**), with Africa having the highest proportion of infants (50%) receiving only human milk (no formula or fortifier) and Latin America the lowest (20%).

## DISCUSSION

This analysis included over 35 000 very preterm or VLBW infants from 12 middle-income countries, making it one of the first and largest of its kind. Our key findings included: a high percent (84%) of infants receiving at least some human milk at NICU discharge/transfer, with 34% receiving exclusively human milk; evidence of slower weight gain and head growth during the NICU hospitalisation relative to a foetal reference; and slower weight gain among infants fed a mixed diet comprising human milk with formula and/or fortifier, compared to formula only.

The WHO strongly recommends mother’s own milk as the standard of care across all countries for infants born very preterm or VLBW, based on mortality benefits as well as protection against NEC–a health benefit specific to this population–and other short- and long-term benefits, such as reduced infection and enhanced neurodevelopment, that apply generally to all newborns [21]. Compared with the USA, where we previously reported ~50% use of any human milk at NICU discharge [22], this study of middle-income countries demonstrated greater adherence (84%) to the WHO recommendation. Our database did not allow us to differentiate between maternal milk and pasteurised donor human milk (‘donor milk’), but given the limited availability of donor milk in many middle-income countries and its infrequent clinical use at the time of hospital discharge, we speculate that most human milk use documented at discharge represented mother’s own milk. An exception may be Brazil, which has a long-standing and well-established network of human milk banks that provide donor milk for hospitalised infants. It is notable that infants who were receiving only human milk at the time of discharge had fewer morbidities, but we are unable to make causal inferences about the effect of diet type on serious acute complications of prematurity, such as NEC, which would have occurred prior to the assessment of diet.

Our study indicated declining weight and head circumference z-scores during the NICU hospitalisation relative to a widely-used foetal reference (Fenton) that is based on birth data from high income countries harmonised with data from the WHO Growth Standard. A mean z-score decline in weight of 1.4 means that the average infant in our cohort left the hospital with a body weight that was 1.4 standard deviations below their birth weight for PMA. Although tracking with the foetus of the same PMA (the ‘reference foetus’) – as recommended by some authors [5,23] – is increasingly recognised to be unachievable [24,25], the change in z-score-for-age from birth to NICU discharge as a growth indicator provides context and facilitates comparison across studies and settings. For context, one reference suggests that a NICU weight z-score change <−0.8 to −1.2 represents mild malnutrition and <−1.2 to 2 represents moderate malnutrition [26]

The declines in weight and head circumference z-scores we observed here are more pronounced than those reported in high income countries. In our prior USA-based study [13], we observed a ~0.9 z-score decline in weight from birth to NICU discharge/transfer. Results from a large study in the UK [24] were similar to those from our USA study and in contrast with the substantially greater 1.4 z-score decline in this middle-income country cohort. Similarly, a South Asian study [26] reported a weight z-score decline of 1.4 in Malaysia and 2.2 in Singapore, and our Brazilian study [27] found a weight z-score decline of 1.9. This magnitude of difference (half standard deviation or greater) is likely to be clinically significant. Head growth, which tracks with brain growth, was also slower in this middle-income country cohort than in cohorts from high income countries, although differences in head growth were less pronounced than differences in weight gain. Further, we did not see any evidence of improvement in growth outcomes over time, in contrast to data from the USA [13]

Weight gain and head growth are driven in large part by dietary intake of nutrients. One approach to enhancing dietary intake of key nutrients, while also maximising benefits of human milk, is through fortification using commercial products that add multiple nutrients while minimally displacing the volume of milk. Use of commercial fortifiers improves short-term weight gain (mean difference *vs*. no fortification = 1.8 g per kg/day), linear growth (mean difference = 0.11 cm/week), and head growth (mean difference = 0.06 cm/week), according to a previous meta-analysis [28]. This practice is widespread in high-resource NICUs [28], but commercial fortifier availability is limited in many low- and middle-income countries where infant formula may be used instead to fortify human milk [7]. Here, 59% of infants receiving human milk at discharge were also receiving formula and/or fortifier (‘mixed’ diet) – a proportion lower than the 88% observed in our USA study [29]. Diet at discharge was associated with weight gain indicators, specifically infants receiving formula only had more favourable outcomes (less weight z-score decline, faster weight gain velocity) than infants receiving a mixed diet. Although firm conclusions are limited, these findings suggest clinically important differences in nutrient delivery between groups that bear further study to inform fortification practices.

The strengths of our study include a standardised dataset collected across many middle-income countries, NICUs, and individual infants – the largest of its kind, focussed specifically on very preterm, VLBW infants. An important limitation, however, is the cross-sectional assessment of diet at discharge/transfer, and not earlier in the hospitalisation, due to available data. Ascertainment of diet at discharge is likely to underestimate the proportion of infants who received any human milk, because mothers who initiated lactation after their very preterm delivery may have struggled to maintain their supply or chosen to stop providing their milk prior to the infant reaching discharge. Infants classified as receiving ‘human milk only’ may have received formula and/or fortifier earlier in the hospitalisation before transitioning to unfortified maternal milk in preparation for discharge. These classification errors are likely to cause underestimation of the growth deficit associated with a ‘human milk only’ diet. The ‘mixed’ diet group is heterogeneous, and we lacked details about milk volumes and proportions necessary to assess a dose-response relationship of milk type with growth or other outcomes.

Other limitations include a small number of infants (~1%) who were excluded from this analysis due to missing data. We lacked data on body length and on neurodevelopmental outcomes, although early weight gain does predict later neurodevelopment [30]. We also acknowledge that measurement variability may exist between sites. Hospitals in Brazil and South Africa accounted for the majority of our sample; generalisability to other countries, especially those underrepresented in our sample, may therefore be limited. For example, practices in a single public NICU in Pakistan may differ substantially from those in more than one hundred private hospitals in South Africa. Although we attempted to address this issue by presenting results stratified by region, we recognise that substantial heterogeneity exists. We did not have access to regional or hospital-level data on contextual factors such as donor milk and fortifier availability or nutrition protocol use that might shape feeding decisions and growth trajectories in this setting. Generalisability may also be limited to hospitals without access to parenteral nutrition. Three quarters of participating hospitals were private, and we acknowledge the limited generalisability to public or other hospital settings. Apart from hospital-level data that virtually all sites had access to parenteral nutrition, we lacked data about specific hospital-level practices related to initiation, advancement, target volumes, and fortification of human milk, and about donor milk availability and requirements of families to pay out-of-pocket for donor milk, formula, and/or fortification. Accuracy of gestational dating may be limited in settings where antenatal ultrasound is not used routinely. We used regression to adjust for many key covariates, but note an important lack of data on social determinants of health and the possibility of residual confounding by these and/or other unmeasured factors.

Because the NICU hospitalisation coincides with a critical period in brain development, optimising nutrition in such contexts can have long-lasting benefits [31,32]. Among possible nutritional interventions, enteral-based strategies are more straightforward to implement than parenteral strategies, particularly when resources are limited. Fortification of human milk is one strategy to protect breastfeeding while also ensuring that protein, energy, and micronutrient intakes meet recommended targets [31]. Feeding higher milk volumes to increase energy and nutrient delivery is an alternative (or complementary) strategy [33], but target intakes of some nutrients, such as protein, calcium, and phosphorus, cannot be met through volume alone. Early establishment of enteral feeding through the use of a standardised guideline is another strategy to improve growth outcomes in lower resource settings [34]. Non-nutritional interventions such as family-integrated care and/or kangaroo mother care [35] may also contribute to improved growth outcomes in these settings, but do not replace adequate nutrient delivery.

## CONCLUSIONS

We observed a high use of human milk in NICUs in middle-income countries, consistent with WHO recommendations. We also found substantially slower weight gain and head growth among infants cared for in these settings, as compared with published data from high income countries, and that using human milk mixed with formula and/or fortifier at discharge was associated with slower weight gain than using formula alone. The health benefits of human milk for small, vulnerable newborns in middle-income countries and the potential harms of using formula routinely in place of maternal milk in this context cannot be overstated. As middle-income countries invest in life-saving care for small, vulnerable newborns, it is important to carefully weigh aspects of NICU care that will enhance long-term outcomes among survivors, including NICU diet. Further evidence from rigorously designed and implemented clinical trials of human milk fortification is urgently needed, as well as from prospective observational studies that quantify the type, dose, and duration of milk fortification. Until more definitive data and recommendations are available regarding routine human milk fortification, clinicians caring for very preterm, VLBW infants in middle-income countries should closely monitor their in-hospital weight gain, linear growth, and head growth. Fortification using locally available products – including commercial multicomponent human milk fortifier or preterm infant formula powder adapted for use as a fortifier – should be prioritised for infants with suboptimal growth.

## Additional material


Online Supplementary Document
