# Supplementary material for: Neonatal diet and growth outcomes in hospitalised very preterm infants: an observational study in middle income countries in Africa, Asia, and Latin America
Source: J Glob Health. 2025 Dec 22;15:04340. doi: 10.7189/jogh.15.04340 (PMC12720493; doi:10.7189/jogh.15.04340)
Supplement: Online Supplementary Document [file jogh-15-04340-s001.pdf]

Supplement to: Belfort M, Ehret D, Greenberg L, Lee A, Procianoy R, Semrau K, Silveira R, Tooke L, Edwards E. Neonatal diet and growth outcomes in hospitalised very preterm infants: an observational study in middle income countries in Africa, Asia, and Latin America. J Glob Health. 2025;15:04340.

Table S1. Number of hospitals and participants and gestational age distribution by region and country

			Gestational age category, completed weeks		
	Hospitals, n	Participants, n	≤26	27-29	30-32
Africa	119	15754	1262 (8%)	7532 (48%)	6960 (44%)
Botswana	2	NA	NA	NA	NA
Namibia	2	NA	NA	NA	NA
South Africa	115	NA	NA	NA	NA
Asia	37	3155	348 (11%)	1316 (42%)	1491 (46%)
China	2	NA	NA	NA	NA
India	30	NA	NA	NA	NA
Malaysia	2	NA	NA	NA	NA
Pakistan	1	NA	NA	NA	NA
Turkey	2	NA	NA	NA	NA
Latin America	66	16934	1678 (10%)	7178 (42%)	8078 (48%)
Argentina	6	NA	NA	NA	NA
Brazil	57	NA	NA	NA	NA
Columbia	2	NA	NA	NA	NA
Mexico	1	NA	NA	NA	NA

NA indicates not applicable; numbers are reported by region only due to small numbers of hospitals in most countries

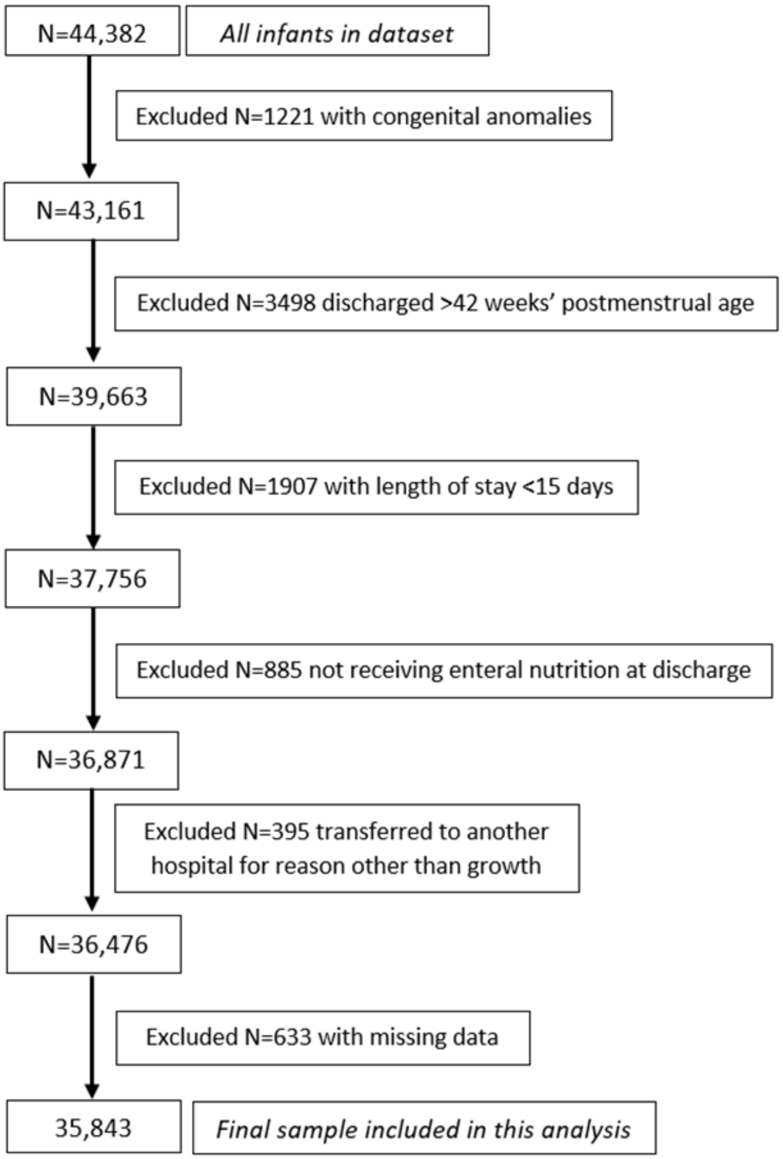

Figure S1. Participant flow.

Table S2. Adjusted associations of diet at discharge or transfer with weight gain and head growth, stratified by gestational age (GA) and fetal growth status (n=35,843)

Growth measure	*Adjusted Mean (SE)*			*Difference (95% CI)*	
	1) Human milk only	2) Mixed	3) Formula only	1 vs. 2	2 vs. 3
GA, completed weeks†					
≤26 (n=3,288)					
Weight z-score change	-2.01 (0.05)	-1.89 (0.05)	-1.70 (0.05)	0.11 (0.03, 0.20)	0.19 (0.12, 0.26)*
Weight gain velocity (g/kg/day)	11.74 (0.14)	11.92 (0.13)	12.44 (0.13)	0.18 (-0.07, 0.43)	0.52 (0.32, 0.71)*
Head z-score change	-1.72 (0.07)	-1.65 (0.06)	-1.61 (0.08)	0.07 (-0.07, 0.21)	0.04 (-0.08, 0.16)
Head growth (cm/week)	0.63 (0.01)	0.65 (0.01)	0.66 (0.01)	0.02 (0.00, 0.04)	0.01 (-0.01, 0.02)
27-29 (n=16,026)					
Weight z-score change	-1.53 (0.04)	-1.45 (0.04)	-1.31 (0.04)	0.08 (-0.03, 0.18)	0.15 (0.09, 0.20)*
Weight gain velocity (g/kg/day)	11.23 (0.17)	11.34 (0.19)	11.96 (0.13)	0.11 (-0.40, 0.62)	0.62 (0.33, 0.91)*
Head z-score change	-1.34 (0.05)	-1.31 (0.05)	-1.23 (0.05)	0.02 (-0.08, 0.13)	0.08 (-0.01, 0.18)
Head growth (cm/week)	0.59 (0.01)	0.60 (0.01)	0.62 (0.01)	0.01 (-0.02, 0.03)	0.02 (0.00, 0.04)
30-32 (n=16,529)					
Weight z-score change	-1.17 (0.03)	-1.09 (0.03)	-0.95 (0.03)	0.08 (0.02, 0.13)	0.14 (0.09, 0.19)*
Weight gain velocity (g/kg/day)	10.80 (0.18)	11.11 (0.20)	11.80 (0.16)	0.31 (-0.12, 0.74)	0.70 (0.36, 1.03)*
Head z-score change	-0.85 (0.04)	-0.80 (0.04)	-0.72 (0.04)	0.05 (-0.01, 0.11)	0.08 (0.00, 0.16)
Head growth (cm/week)	0.57 (0.01)	0.59 (0.01)	0.60 (0.01)	0.02 (0.00, 0.03)	0.02 (0.00, 0.04)
Fetal growth status‡					
Small for gestational age (n=4,566)					
Weight z-score change	-1.24 (0.04)	-1.18 (0.04)	-1.08 (0.04)	0.05 (-0.04, 0.14)	0.11 (0.05, 0.17)*
Weight gain velocity (g/kg/day)	12.98 (0.21)	13.08 (0.20)	13.61 (0.18)	0.10 (-0.39, 0.59)	0.53 (0.26, 0.80)*
Head z-score change	-0.59 (0.06)	-0.52 (0.05)	-0.48 (0.07)	0.07 (-0.04, 0.19)	0.04 (-0.10, 0.18)
Head growth (cm/week)	0.64 (0.01)	0.66 (0.01)	0.67 (0.01)	0.02 (0.00, 0.04)	0.01 (-0.02, 0.03)
Not small for gestational age (n=31,277)					
Weight z-score change	-1.42 (0.03)	-1.34 (0.04)	-1.18 (0.03)	0.08 (0.01, 0.15)	0.15 (0.11, 0.20)*
Weight gain velocity (g/kg/day)	10.82 (0.16)	11.05 (0.19)	11.71 (0.14)	0.24 (-0.22, 0.69)	0.66 (0.36, 0.95)*
Head z-score change	-1.22 (0.04)	-1.21 (0.04)	-1.12 (0.04)	0.02 (-0.05, 0.08)	0.09 (0.02, 0.16)
Head growth (cm/week)	0.58 (0.01)	0.58 (0.01)	0.60 (0.01)	0.01 (-0.01, 0.02)	0.02 (0.00, 0.04)

Mixed diet indicates human milk plus formula or fortifier. Small for gestational age defined as birth weight below the tenth percentile on the Fenton reference . Estimates adjusted for sex, gestational age at birth, birth weight z-score, postmenstrual age at discharge, NICU morbidity, hospital.

*Difference is statistically significant after Bonferroni correction (p <0.005).

†diet*GA interaction p-values: weight z-score change, 0.005; weight gain velocity, <0.001; head z-score change, 0.86; head growth, 0.32

‡diet*fetal growth interaction p-values: weight z-score change, <0.001; weight gain velocity, <0.001; head z-score change, <0.001; head growth, <0.001

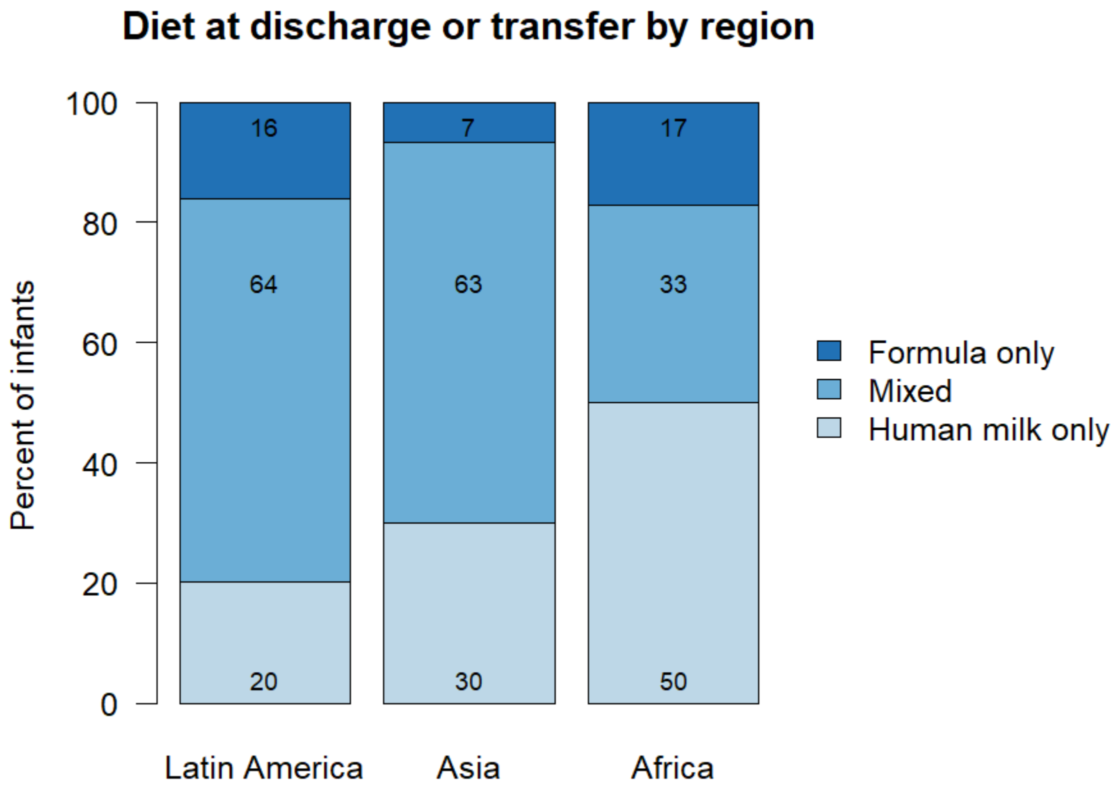

Figure S2. Diet at discharge or transfer by region. The percent of infants receiving human milk only (no formula or fortifier) was highest in Africa (50%) and lowest in Latin America (20%).

Table S3. Vermont Oxford Network member hospitals who contributed the data used in this study

Hospital Name	City	Country
Clinica y Maternidad Suizo Argentina	Buenos Aires	Argentina
Hospital Italiano De Buenos Aires	Buenos Aires	Argentina
Sanatorio Otamendi	Buenos Aires	Argentina
Sanatorio Trinidad Palermo	Buenos Aires	Argentina
Sanatorio Trinidad Ramos Mejia	Ramos Mejia	Argentina
Sanatorio Trinidad San Isidro	San Isidro	Argentina
Lenmed Bokamoso Private Hospital	Gaborone	Botswana
Life Gaborone Private Hospital	Gaborone	Botswana
Hospital das ClÃnicas / UFMG	Belo Horizonte	Brazil
Hospital Sofia Feldman	Belo Horizonte	Brazil
Maternidade Escola Hilda BrandÃ£o / FCMMG	Belo Horizonte	Brazil
Neocenter S.A.	Belo Horizonte	Brazil
Hospital Santo Antonio	Blumenau	Brazil
Hosp das ClÃnicas /Faculdade de Med de Botucatu	Botucatu	Brazil
Hospital Materno Infantil de Brasilia	Brasilia	Brazil
Centro de AtenÃ§Ã£o a SaÃºde da Mulher / UNICAMP	Campinas	Brazil
CEPLIN Instituto de Neonatologia e Pediatria Ltda	Campos dos Goytacazes	Brazil
Hospital Universitario ULBRA/GAMP - Canoas	Canoas RS	Brazil
Hospital Geral de Caxias do Sul	Caxias do Sul	Brazil
Hospital Unimed Caxias do Sul	Caxias do Sul	Brazil
Hospital das ClÃnicas / UFPR	Curitiba	Brazil
Hospital Estadual de Diadema / UNIFESP	Diadema	Brazil
Clinica Santa Helena LTDA	Florianopolis	Brazil
Maternidade Carmela Dutra	Florianopolis	Brazil
Hospital Maternidade Marieta Konder Bornhausen	Itajai	Brazil
Maternidade Darcy Vargas	Joinville	Brazil
Hospital UniversitÃ¡rio / UEL	Londrina	Brazil
UTI Neonatal Nicola Albano Ltda	Macae	Brazil
Maternidade Escola Januario Cicco	Natal	Brazil
Hospital Universitario Antonio Pedro	Niteroi	Brazil
Casa De Saude Laranjeiras Filial Icarai	Niteroi RJ	Brazil
Santa Casa de Misericordia de Passos	Passos	Brazil
Hospital de Clinicas de Porto Alegre	Porto Alegre	Brazil
Hospital Moinhos de Vento	Porto Alegre	Brazil
Hospital SÃ£o Lucas da PUCRS	Porto Alegre	Brazil
Irmandade Santa Casa de Misericordia	Porto Alegre	Brazil
Hospital Agamenon Magalhaes	Recife	Brazil
Instituto de Medicina Integral Prof Fernando Figue	Recife	Brazil
Hosp. das ClÃnicas de RibeirÃ£o Preto/USP	Ribeirao Preto	Brazil
Clinica Perinatal Gloria	Rio de Janeiro	Brazil
Hospital Univers. Pedro Ernesto/UERJ	Rio de Janeiro	Brazil
Instituto Fernandes Figueira / Fiocruz	Rio de Janeiro	Brazil
Oeste D'Or - Rede D'Or - Sao Luiz	Rio de Janeiro	Brazil
Perinatal Clinic - Barra da Tijuca Branch	Rio de Janeiro	Brazil

Universidade Federal do Rio de Janeiro	Rio de Janeiro	Brazil
Hospital Regional Alto Vale	Rio do Sul	Brazil
Hospital Portugues - Neomed	Salvador	Brazil
Hospital Santo Amaro	Salvador	Brazil
Maternidade Jose Maria de Magalhaes Netto	Salvador	Brazil
Hospital Municipal Universitario de Sao Bernardo	Sao Bernardo do Campo	Brazil
Hospital da Crianca e Maternidade (HCM)	Sao Jose do Rio Preto	Brazil
Hospital UniversitÃ¡rio / UFMA	Sao Luis	Brazil
Maternidade De Alta Complexidade Do MaranhÃ£o	Sao Luis	Brazil
Hospital e Maternidade Santa Joana	Sao Paulo	Brazil
Hospital e Maternidade Sao Luiz	Sao Paulo	Brazil
Hospital e Maternidade Sepaco	Sao Paulo	Brazil
Hospital Israelita Albert Einstein	Sao Paulo	Brazil
Hospital Municipal Vila Santa Catarina	Sao Paulo	Brazil
Hospital SÃ£o Paulo / Escola Paulista de Medicina â€“	Sao Paulo	Brazil
Hospital UniversitÃ¡rio / USP	Sao Paulo	Brazil
Instituto da CrianÃ§a do Hospital das ClÃ-nicas -Fac	Sao Paulo	Brazil
Pro Matre Paulista	Sao Paulo	Brazil
Hospital Estadual SumarÃ© / UNICAMP	Sumare	Brazil
Hospital Geral de Pirajussara / UNIFESP	Taboao da Serra - SP	Brazil
Hospital das ClÃ-nicas / UFU	Uberlandia	Brazil
Queen Mary Hospital	Hong Kong	China
Bao'an Maternal & Child Healthcare Hospital	Shenzhen	China
Clinica Del Prado Grupo Quiron Salud	Medellin	Colombia
Hospital Universitario San Vicente de Paul	Medellin	Colombia
Cloudnine Bellandur	Bangalore	India
Cloudnine Hospital Electronic City	Bangalore	India
Cloudnine Hospital Kanakapura	Bangalore	India
Cloudnine Hospital Sahakar Nagar	Bangalore	India
Cloudnine HRBR	Bangalore	India
Cloudnine Jayanagar	Bangalore	India
Cloudnine Malleshwaram	Bangalore	India
Cloudnine Old Airport Road	Bangalore	India
Cloudnine Hospital Sarjapur	Bangalore, Karnataka	India
Kids Clinic India Limited Cloudnine Hospital	Bangalore, Karnataka	India
Cloudnine Whitefield	Bengaluru	India
Cloudnine Chandigarh	Chandigarh	India
Cloudnine Chennai	Chennai	India
Cloudnine Hospital Old Mahabalipuram Road	Chennai	India
Cloudnine Hospital East Delhi	East Delhi	India
Cloudnine Hospital Faridabad	Faridabad	India
Cloudnine Gurgaon	Gurgaon	India
Cloudnine Old Gurgaon Sector 14 Hospital	Gurgaon	India
Cloudnine Hospital Golf Course Road	Gurugram	India
Cloudnine Hitech City	Hyderabad	India
The Birthplace by Cloudnine Hospital	Hyderabad	India
Cloudnine Malad	Mumbai	India

Cloudnine Vashi	Navi Mumbai	India
Cloudnine Hospital Nerul	Nerul, Navi Mumbai, Maharashtra	India
Cloudnine Hospital Kailash Colony	New Delhi	India
Cloudnine Hospital Punjabi Bagh	New Delhi	India
Cloudnine Hospital Noida	Noida	India
Cloudnine Hospital Panchkula	Panchkula	India
Cloudnine Kalyani Nagar Pune	Pune	India
Cloudnine Shivajinagar Pune	Pune	India
University Kebangsaan Malaysia	Kuala Lumpur	Malaysia
University Malaya Medical Centre	Kuala Lumpur	Malaysia
Instituto Nacional de Perinatologia Isidro Espinos	Cuidad de Mexico	Mexico
Mediclinic Swakopmund	Swakopmund	Namibia
Mediclinic Windhoek	Windhoek	Namibia
University of Child Health Sciences	Lahore	Pakistan
Netcare Akasia Hospital	Akasia	South Africa
Netcare Alberton Hospital	Alberton	South Africa
Netcare Alberlito Hospital	Ballito	South Africa
Mediclinic Louis Leipoldt	Bellville	South Africa
Netcare Lakeview Hospital	Benoni	South Africa
Netcare Linmed Hospital	Benoni	South Africa
Life The Glynnwood Hospital	Benoni	South Africa
Mediclinic Hoogland	Bethlehem	South Africa
Life Rosepark Hospital	Bloemfontein	South Africa
Mediclinic Bloemfontein	Bloemfontein	South Africa
Netcare Pelonomi Private Hospital	Bloemfontein	South Africa
Netcare Sunward Park Hospital	Boksburg/Gauteng	South Africa
Life Dalview Hospital	Brakpan	South Africa
Mediclinic Sandton	Bryanston	South Africa
Mediclinic Cape Gate	Cape Gate	South Africa
Groote Schuur Hospital	Cape Town	South Africa
Life Kingsbury Hospital	Cape Town	South Africa
Life Vincent Pallotti Hospital	Cape Town	South Africa
Mediclinic Cape Town	Cape Town	South Africa
Mediclinic Milnerton	Cape Town	South Africa
Mowbray Maternity Hospital	Cape Town	South Africa
Netcare Christiaan Barnard Memorial Hospital	Cape Town	South Africa
Netcare Kuils River Hospital	Cape Town	South Africa
New Somerset Hospital	Cape Town	South Africa
Tygerberg Hospital	Cape Town	South Africa
Joint Medical Holdings City Hospital PMG	Durban	South Africa
King Edward Hospital	Durban	South Africa
Lenmed Ethekwini Hospital and Heart Centre	Durban	South Africa
Lenmed Shifa Private Hospital	Durban	South Africa
Netcare Kingsway Hospital	Durban	South Africa
Netcare Parklands Hospital	Durban	South Africa
Netcare St. Augustines Hospital	Durban	South Africa
Netcare Umhlanga Hospital	Durban	South Africa

Life Beacon Bay Hospital	East London	South Africa
Life Empangeni Hospital	Empangeni	South Africa
Mediclinic Kloof	Erasmuskloof	South Africa
Life Flora Hospital	Florida	South Africa
Life Suikerbosrand Hospital	Gauteng	South Africa
Mediclinic George	George	South Africa
Lenmed Ahmed Kathrada Private Hospital	Johannesburg	South Africa
Life Bedford Gardens Hospital	Johannesburg	South Africa
Life Brenthurst Hospital	Johannesburg	South Africa
Life Fourways Hospital	Johannesburg	South Africa
Netcare Linkwood Hospital	Johannesburg	South Africa
Netcare Mulbarton Hospital	Johannesburg	South Africa
Netcare Park Lane Clinic	Johannesburg	South Africa
Netcare Pinehaven Hospital	Johannesburg	South Africa
Netcare Waterfall City Hospital	Johannesburg	South Africa
Charlotte Maxeke Johannesburg Academic Hospital	Johannesburg Gauteng	South Africa
Arwyp Medical Centre	Kempton Park	South Africa
Lenmed Royal Hospital and Heart Centre	Kimberley	South Africa
Mediclinic Kimberley	Kimberley	South Africa
Life Anncron Hospital	Klerksdorp	South Africa
Netcare Kroon Hospital	Kroonstad	South Africa
Netcare Krugersdorp Hospital	Krugersdorp	South Africa
Lenmed La Verna Private Hospital	Ladysmith	South Africa
Life Eugene Marais Hospital	Les Marais	South Africa
Mediclinic Limpopo	Limpopo	South Africa
Mediclinic Legae	Mabopane	South Africa
Netcare Margate Hospital	Margate	South Africa
Netcare Garden City Clinic	Mayfair West	South Africa
Life Midmed Hospital	Middelburg	South Africa
Life Carstenhof Clinic	Midrand	South Africa
Mediclinic Midstream	Midstream	South Africa
Netcare Blaauwberg Hospital	Milnerton	South Africa
Life Mount Edgecombe Hospital	Mount Edgecombe	South Africa
Netcare N1 City Hospital	N1 City	South Africa
Mediclinic Nelspruit	Nelspruit	South Africa
Mediclinic Newcastle	Newcastle	South Africa
Mediclinic Paarl	Paarl	South Africa
Mediclinic Panorama	Panorama	South Africa
Mediclinic Pietermaritzburg	Pietermaritzburg	South Africa
Netcare St. Anneâ€™s Hospital	Pietermaritzburg	South Africa
Life The Crompton Hospital	Pinetown	South Africa
Mediclinic Constantiaberg	Plumstead	South Africa
Life Mercantile Hospital	Port Elizabeth	South Africa
Life St. George's Hospital	Port Elizabeth	South Africa
Netcare Greenacres Hospital	Port Elizabeth	South Africa
Mediclinic Potchefstroom	Potchefstroom	South Africa
Dr. George Mukhari Academic Hospital	Pretoria	South Africa

Life Wilgers Hospital	Pretoria	South Africa
Mediclinic Medforum	Pretoria	South Africa
Mediclinic Muelmed	Pretoria	South Africa
Netcare Femina Hospital	Pretoria	South Africa
Netcare Montana Hospital	Pretoria	South Africa
Netcare Pretoria East Hospital	Pretoria	South Africa
Netcare Unitas Hospital	Pretoria	South Africa
Steve Biko Academic Hospital	Pretoria	South Africa
Tembisa Provincial Tertiary Hospital (TPTH)	Pretoria	South Africa
Life Roseacres Hospital	Primrose	South Africa
Netcare Olivedale Clinic	Randburg	South Africa
Lenmed Randfontein Private Hospital	Randfontein	South Africa
Life Healthcare Robinson Private Hospital	Randfontein	South Africa
Netcare The Bay Hospital	Richards Bay	South Africa
Life Wilgeheuwel Hospital	Roodepoort	South Africa
Life Peglerae Hospital	Rustenburg	South Africa
Netcare Ferncrest Hospital	Rustenburg	South Africa
Mediclinic Morningside	Sandton	South Africa
Netcare Sunninghill Hospital	Sandton	South Africa
Netcare Pholoso Hospital	Savannah, Polokwane	South Africa
Mediclinic Vergelegen	Somerset West	South Africa
Life Springs Parkland Hospital	Springs	South Africa
Netcare N17 Hospital	Springs Gauteng	South Africa
Mediclinic Stellenbosch	Stellenbosch	South Africa
Lenmed Zamokuhle Private Hospital	Tembisa	South Africa
Mediclinic Victoria	Tongaat	South Africa
Mediclinic Highveld	Trichardt	South Africa
Mediclinic Tzaneen	Tzaneen	South Africa
Netcare Cuyler Hospital	Ultenhage East Cape	South Africa
Mediclinic Emfuleni	Vanderbiylpark	South Africa
Mediclinic Vereeniging	Vereeniging	South Africa
Mediclinic Welkom	Welkom	South Africa
Life Westville Hospital	Westville	South Africa
Life Cosmos Hospital	Witbank	South Africa
Mediclinic Worcester	Worcester	South Africa
Gazi University Hospital	Ankara	Turkey
Cerrahpasa Medical Faculty, Neonatology Unit	Istanbul	Turkey
Hospital Name	City	Country
Clinica y Maternidad Suizo Argentina	Buenos Aires	Argentina
Hospital Italiano De Buenos Aires	Buenos Aires	Argentina
Sanatorio Otamendi	Buenos Aires	Argentina
Sanatorio Trinidad Palermo	Buenos Aires	Argentina
Sanatorio Trinidad Ramos Mejia	Ramos Mejia	Argentina
Sanatorio Trinidad San Isidro	San Isidro	Argentina
Lenmed Bokamoso Private Hospital	Gaborone	Botswana
Life Gaborone Private Hospital	Gaborone	Botswana
Hospital das ClÃ-nicas / UFMG	Belo Horizonte	Brazil

Hospital Sofia Feldman	Belo Horizonte	Brazil
Maternidade Escola Hilda BrandÃ£o / FCMMG	Belo Horizonte	Brazil
Neocenter S.A.	Belo Horizonte	Brazil
Hospital Santo Antonio	Blumenau	Brazil
Hosp das ClÃ-nicas /Faculdade de Med de Botucatu	Botucatu	Brazil
Hospital Materno Infantil de Brasilia	Brasilia	Brazil
Centro de AtenÃ§Ã£o a SaÃºde da Mulher / UNICAMP	Campinas	Brazil
CEPLIN Instituto de Neonatologia e Pediatria Ltda	Campos dos Goytacazes	Brazil
Hospital Universitario ULBRA/GAMP - Canoas	Canoas RS	Brazil
Hospital Geral de Caxias do Sul	Caxias do Sul	Brazil
Hospital Unimed Caxias do Sul	Caxias do Sul	Brazil
Hospital das ClÃ-nicas / UFPR	Curitiba	Brazil
Hospital Estadual de Diadema / UNIFESP	Diadema	Brazil
Clinica Santa Helena LTDA	Florianopolis	Brazil
Maternidade Carmela Dutra	Florianopolis	Brazil
Hospital Maternidade Marieta Konder Bornhausen	Itajai	Brazil
Maternidade Darcy Vargas	Joinville	Brazil
Hospital UniversitÃ¡rio / UEL	Londrina	Brazil
UTI Neonatal Nicola Albano Ltda	Macae	Brazil
Maternidade Escola Januario Cicco	Natal	Brazil
Hospital Universitario Antonio Pedro	Niteroi	Brazil
Casa De Saude Laranjeiras Filial Icarai	Niteroi RJ	Brazil
Santa Casa de Misericordia de Passos	Passos	Brazil
Hospital de Clinicas de Porto Alegre	Porto Alegre	Brazil
Hospital Moinhos de Vento	Porto Alegre	Brazil
Hospital SÃ£o Lucas da PUCRS	Porto Alegre	Brazil
Irmandade Santa Casa de Misericordia	Porto Alegre	Brazil
Hospital Agamenon Magalhaes	Recife	Brazil
Instituto de Medicina Integral Prof Fernando Figue	Recife	Brazil
Hosp. das ClÃ-nicas de RibeirÃ£o Preto/USP	Ribeirao Preto	Brazil
Clinica Perinatal Gloria	Rio de Janeiro	Brazil
Hospital Univers. Pedro Ernesto/UERJ	Rio de Janeiro	Brazil
Instituto Fernandes Figueira / Fiocruz	Rio de Janeiro	Brazil
Oeste D'Or - Rede D'Or - Sao Luiz	Rio de Janeiro	Brazil
Perinatal Clinic - Barra da Tijuca Branch	Rio de Janeiro	Brazil
Universidade Federal do Rio de Janeiro	Rio de Janeiro	Brazil
Hospital Regional Alto Vale	Rio do Sul	Brazil
Hospital Portugues - Neomed	Salvador	Brazil
Hospital Santo Amaro	Salvador	Brazil
Maternidade Jose Maria de Magalhaes Netto	Salvador	Brazil
Hospital Municipal Universitario de Sao Bernardo	Sao Bernardo do Campo	Brazil
Hospital da Crianca e Maternidade (HCM)	Sao Jose do Rio Preto	Brazil
Hospital UniversitÃ¡rio / UFMA	Sao Luis	Brazil
Maternidade De Alta Complexidade Do MaranhÃ£o	Sao Luis	Brazil
Hospital e Maternidade Santa Joana	Sao Paulo	Brazil
Hospital e Maternidade Sao Luiz	Sao Paulo	Brazil
Hospital e Maternidade Sepaco	Sao Paulo	Brazil

Hospital Israelita Albert Einstein	Sao Paulo	Brazil
Hospital Municipal Vila Santa Catarina	Sao Paulo	Brazil
Hospital SÃ£o Paulo / Escola Paulista de Medicina â€“	Sao Paulo	Brazil
Hospital UniversitÃ¡rio / USP	Sao Paulo	Brazil
Instituto da CrianÃ§a do Hospital das ClÃ-nicas -Fac	Sao Paulo	Brazil
Pro Matre Paulista	Sao Paulo	Brazil
Hospital Estadual SumarÃ© / UNICAMP	Sumare	Brazil
Hospital Geral de Pirajussara / UNIFESP	Taboao da Serra - SP	Brazil
Hospital das ClÃ-nicas / UFU	Uberlandia	Brazil
Queen Mary Hospital	Hong Kong	China
Bao'an Maternal & Child Healthcare Hospital	Shenzhen	China
Clinica Del Prado Grupo Quiron Salud	Medellin	Colombia
Hospital Universitario San Vicente de Paul	Medellin	Colombia
Cloudnine Bellandur	Bangalore	India
Cloudnine Hospital Electronic City	Bangalore	India
Cloudnine Hospital Kanakapura	Bangalore	India
Cloudnine Hospital Sahakar Nagar	Bangalore	India
Cloudnine HRBR	Bangalore	India
Cloudnine Jayanagar	Bangalore	India
Cloudnine Malleshwaram	Bangalore	India
Cloudnine Old Airport Road	Bangalore	India
Cloudnine Hospital Sarjapur	Bangalore, Karnataka	India
Kids Clinic India Limited Cloudnine Hospital	Bangalore, Karnataka	India
Cloudnine Whitefield	Bengaluru	India
Cloudnine Chandigarh	Chandigarh	India
Cloudnine Chennai	Chennai	India
Cloudnine Hospital Old Mahabalipuram Road	Chennai	India
Cloudnine Hospital East Delhi	East Delhi	India
Cloudnine Hospital Faridabad	Faridabad	India
Cloudnine Gurgaon	Gurgaon	India
Cloudnine Old Gurgaon Sector 14 Hospital	Gurgaon	India
Cloudnine Hospital Golf Course Road	Gurugram	India
Cloudnine Hitech City	Hyderabad	India
The Birthplace by Cloudnine Hospital	Hyderabad	India
Cloudnine Malad	Mumbai	India
Cloudnine Vashi	Navi Mumbai	India
Cloudnine Hospital Nerul	Nerul, Navi Mumbai, Maharashtra	India
Cloudnine Hospital Kailash Colony	New Delhi	India
Cloudnine Hospital Punjabi Bagh	New Delhi	India
Cloudnine Hospital Noida	Noida	India
Cloudnine Hospital Panchkula	Panchkula	India
Cloudnine Kalyani Nagar Pune	Pune	India
Cloudnine Shivajinagar Pune	Pune	India
University Kebangsaan Malaysia	Kuala Lumpur	Malaysia
University Malaya Medical Centre	Kuala Lumpur	Malaysia
Instituto Nacional de Perinatologia Isidro Espinos	Cuidad de Mexico	Mexico
Mediclinic Swakopmund	Swakopmund	Namibia

Mediclinic Windhoek	Windhoek	Namibia
University of Child Health Sciences	Lahore	Pakistan
Netcare Akasia Hospital	Akasia	South Africa
Netcare Alberton Hospital	Alberton	South Africa
Netcare Alberlito Hospital	Ballito	South Africa
Mediclinic Louis Leipoldt	Bellville	South Africa
Netcare Lakeview Hospital	Benoni	South Africa
Netcare Linmed Hospital	Benoni	South Africa
Life The Glynnwood Hospital	Benoni	South Africa
Mediclinic Hoogland	Bethlehem	South Africa
Life Rosepark Hospital	Bloemfontein	South Africa
Mediclinic Bloemfontein	Bloemfontein	South Africa
Netcare Pelonomi Private Hospital	Bloemfontein	South Africa
Netcare Sunward Park Hospital	Boksburg/Gauteng	South Africa
Life Dalview Hospital	Brakpan	South Africa
Mediclinic Sandton	Bryanston	South Africa
Mediclinic Cape Gate	Cape Gate	South Africa
Groote Schuur Hospital	Cape Town	South Africa
Life Kingsbury Hospital	Cape Town	South Africa
Life Vincent Pallotti Hospital	Cape Town	South Africa
Mediclinic Cape Town	Cape Town	South Africa
Mediclinic Milnerton	Cape Town	South Africa
Mowbray Maternity Hospital	Cape Town	South Africa
Netcare Christiaan Barnard Memorial Hospital	Cape Town	South Africa
Netcare Kuils River Hospital	Cape Town	South Africa
New Somerset Hospital	Cape Town	South Africa
Tygerberg Hospital	Cape Town	South Africa
Joint Medical Holdings City Hospital PMG	Durban	South Africa
King Edward Hospital	Durban	South Africa
Lenmed Ethekwini Hospital and Heart Centre	Durban	South Africa
Lenmed Shifa Private Hospital	Durban	South Africa
Netcare Kingsway Hospital	Durban	South Africa
Netcare Parklands Hospital	Durban	South Africa
Netcare St. Augustines Hospital	Durban	South Africa
Netcare Umhlanga Hospital	Durban	South Africa
Life Beacon Bay Hospital	East London	South Africa
Life Empangeni Hospital	Empangeni	South Africa
Mediclinic Kloof	Erasmuskloof	South Africa
Life Flora Hospital	Florida	South Africa
Life Suikerbosrand Hospital	Gauteng	South Africa
Mediclinic George	George	South Africa
Lenmed Ahmed Kathrada Private Hospital	Johannesburg	South Africa
Life Bedford Gardens Hospital	Johannesburg	South Africa
Life Brenthurst Hospital	Johannesburg	South Africa
Life Fourways Hospital	Johannesburg	South Africa
Netcare Linkwood Hospital	Johannesburg	South Africa
Netcare Mulbarton Hospital	Johannesburg	South Africa

Netcare Park Lane Clinic	Johannesburg	South Africa
Netcare Pinehaven Hospital	Johannesburg	South Africa
Netcare Waterfall City Hospital	Johannesburg	South Africa
Charlotte Maxeke Johannesburg Academic Hospital	Johannesburg Gauteng	South Africa
Arwyp Medical Centre	Kempton Park	South Africa
Lenmed Royal Hospital and Heart Centre	Kimberley	South Africa
Mediclinic Kimberley	Kimberley	South Africa
Life Anncron Hospital	Klerksdorp	South Africa
Netcare Kroon Hospital	Kroonstad	South Africa
Netcare Krugersdorp Hospital	Krugersdorp	South Africa
Lenmed La Verna Private Hospital	Ladysmith	South Africa
Life Eugene Marais Hospital	Les Marais	South Africa
Mediclinic Limpopo	Limpopo	South Africa
Mediclinic Legae	Mabopane	South Africa
Netcare Margate Hospital	Margate	South Africa
Netcare Garden City Clinic	Mayfair West	South Africa
Life Midmed Hospital	Middelburg	South Africa
Life Carstenhof Clinic	Midrand	South Africa
Mediclinic Midstream	Midstream	South Africa
Netcare Blaauwberg Hospital	Milnerton	South Africa
Life Mount Edgecombe Hospital	Mount Edgecombe	South Africa
Netcare N1 City Hospital	N1 City	South Africa
Mediclinic Nelspruit	Nelspruit	South Africa
Mediclinic Newcastle	Newcastle	South Africa
Mediclinic Paarl	Paarl	South Africa
Mediclinic Panorama	Panorama	South Africa
Mediclinic Pietermaritzburg	Pietermaritzburg	South Africa
Netcare St. Anneâ€™s Hospital	Pietermaritzburg	South Africa
Life The Crompton Hospital	Pinetown	South Africa
Mediclinic Constantiaberg	Plumstead	South Africa
Life Mercantile Hospital	Port Elizabeth	South Africa
Life St. George's Hospital	Port Elizabeth	South Africa
Netcare Greenacres Hospital	Port Elizabeth	South Africa
Mediclinic Potchefstroom	Potchefstroom	South Africa
Dr. George Mukhari Academic Hospital	Pretoria	South Africa
Life Wilgers Hospital	Pretoria	South Africa
Mediclinic Medforum	Pretoria	South Africa
Mediclinic Muelmed	Pretoria	South Africa
Netcare Femina Hospital	Pretoria	South Africa
Netcare Montana Hospital	Pretoria	South Africa
Netcare Pretoria East Hospital	Pretoria	South Africa
Netcare Unitas Hospital	Pretoria	South Africa
Steve Biko Academic Hospital	Pretoria	South Africa
Tembisa Provincial Tertiary Hospital (TPTH)	Pretoria	South Africa
Life Roseacres Hospital	Primrose	South Africa
Netcare Olivedale Clinic	Randburg	South Africa
Lenmed Randfontein Private Hospital	Randfontein	South Africa

Life Healthcare Robinson Private Hospital	Randfontein	South Africa
Netcare The Bay Hospital	Richards Bay	South Africa
Life Wilgeheuwel Hospital	Roodepoort	South Africa
Life Peglerae Hospital	Rustenburg	South Africa
Netcare Ferncrest Hospital	Rustenburg	South Africa
Mediclinic Morningside	Sandton	South Africa
Netcare Sunninghill Hospital	Sandton	South Africa
Netcare Pholoso Hospital	Savannah, Polokwane	South Africa
Mediclinic Vergelegen	Somerset West	South Africa
Life Springs Parkland Hospital	Springs	South Africa
Netcare N17 Hospital	Springs Gauteng	South Africa
Mediclinic Stellenbosch	Stellenbosch	South Africa
Lenmed Zamokuhle Private Hospital	Tembisa	South Africa
Mediclinic Victoria	Tongaat	South Africa
Mediclinic Highveld	Trichardt	South Africa
Mediclinic Tzaneen	Tzaneen	South Africa
Netcare Cuyler Hospital	Ultenhage East Cape	South Africa
Mediclinic Emfuleni	Vanderbiylpark	South Africa
Mediclinic Vereeniging	Vereeniging	South Africa
Mediclinic Welkom	Welkom	South Africa
Life Westville Hospital	Westville	South Africa
Life Cosmos Hospital	Witbank	South Africa
Mediclinic Worcester	Worcester	South Africa
Gazi University Hospital	Ankara	Turkey
Cerrahpasa Medical Faculty, Neonatology Unit	Istanbul	Turkey
